# Erbium-Doped Yttrium Aluminium Garnet (Er:YAG) Lasers in the Treatment of Peri-Implantitis

**DOI:** 10.7759/cureus.78279

**Published:** 2025-01-31

**Authors:** Hani Almoharib

**Affiliations:** 1 Department of Periodontics and Community Dentistry, College of Dentistry, King Saud University, Riyadh, SAU

**Keywords:** biofilm removal, dental implants, er:yag laser, peri-implantitis, tissue healing

## Abstract

Erbium-doped yttrium-aluminum-garnet (Er:YAG) lasers have emerged as a promising tool for the treatment of peri-implantitis, a pathological condition characterized by inflammation and bone loss around dental implants. Peri-implantitis, often associated with bacterial biofilms, leads to significant clinical complications, including pocket depth, bleeding, and pain. While traditional treatments such as mechanical debridement have shown limited effectiveness, Er:YAG lasers, with their high absorption by water, are believed to offer enhanced bacterial decontamination and tissue healing through photothermal effects. Studies indicate that Er:YAG lasers can reduce probing depths and improve biofilm removal, though the effects on clinical attachment levels and bone regeneration remain inconsistent. The use of Er:YAG lasers in peri-implantitis treatment is not without challenges, including risks of heat-related tissue damage, undertreatment, and technical limitations such as insufficient penetration into intricate implant surfaces. Additionally, the lack of standardized treatment protocols complicates the widespread adoption of this technology. Research highlights potential improvements when Er:YAG lasers are combined with other modalities, such as antimicrobial photodynamic therapy (aPDT) and ultrasonic devices. Future advancements in laser technology, such as flexible fibers and optimized energy settings, may enhance their clinical application. Despite the promising results, further large-scale randomized controlled trials with extended follow-up periods are required to confirm the long-term benefits of Er:YAG lasers in peri-implantitis management, especially in terms of bone regeneration and bacterial control.

## Introduction and background

Osseointegrated dental implants are now widely utilized to restore missing teeth to functional status [1]. These implants develop bacterial biofilms, which result in inflammatory disorders. Peri-implantitis is a pathological disease that affects the tissues surrounding dental implants. It is marked by inflammation of the peri-implant mucosa and gradual loss of supporting bone [2]. As the number of dental implants worldwide rises, peri-implantitis has become an increasingly concerning pathological condition for dental professionals [3].

Most of the time, peri-implant pathology is asymptomatic. The majority of implants (90%) are regarded as healthy, independent of their actual health state. Major symptoms include pain, bleeding, suppuration, and swelling [4]. Of 458 studied dental implants, Derks et al. (2014) found that 7.6% reported pain, 13.1% reported spontaneous discomfort, 14.6% experienced bleeding, 2% had suppuration history, 5% had swelling, and 15.7% had discomfort when brushing [5].

Prevalence rates ranging from 1% to 47% were reported [6]. The incidence of peri-implantitis increases from 0.4 to 43.9% in three to five years. Meta-analyses yielded weighted mean values ranging from 20% to 22% [7]. In a seven-year study conducted by Ahn et al. (2019) 39.7% of assessed patients had peri-implant mucositis and 16.7% had peri-implant mucositis and peri-implantitis [7]. The 7-year and 10-year survival were 95.87% and 92.6%, respectively, and they followed a similar trend to those seen in other investigations [8].

Difference between peri-implantitis and peri-mucositis

While peri-implantitis is characterized by both the loss of supporting bone and inflammation in the soft tissues surrounding an osseointegrated implant, peri-implant mucositis is biofilm-induced inflammation confined to the soft tissues, without any indication of supporting bone loss [9, 10]. Peri-implantitis also shows a peri-implant pocket ≥4 mm, possibly suppuration, and a distinctive "saucer-" or "crater shaped" bone destruction surrounding the implant, whereas mucositis is characterized by inflammation, erythema, and bleeding of the tissue, especially during examination [11].

Importance of early diagnosis and effective treatment

The long-term effectiveness of dental implant therapy and prevention of peri-implantitis necessitates the early identification and prompt treatment of peri-implant disorders. The risk of developing periodontitis or peri-implantitis can be effectively decreased by early identification of gingivitis or mucositis, respectively. The evaluation of biomarkers may enable the early detection of peri-implant disorders and the rate at which they advance. Once found, periodontal and peri-implant tissue degradation indicators may notify clinicians before clinical symptoms appear. While peri-implantitis detected with more advanced bone loss typically necessitates surgical intervention, early diagnosis of peri-implant illness supports a more conservative non-surgical treatment strategy [12].

Removing the infection and halting bone loss around the implant are the main objectives of peri-implantitis treatment. Non-surgical therapy for peri-implantitis is typically insufficient. However, it serves as the initial course of treatment. Effective therapy requires proper plaque management for dental biofilm prevention, regardless of the opted surgical treatment strategy. Regular examinations are crucial for the long-term care of implants because they enable the dentist to identify and address early indications of peri-implant inflammation that the patient might miss. It must be stated that prevention of peri-implantitis starts at the moment of implant installation [13].

Radiographs and clinical examinations are required to diagnose peri-implant health and disorders. Therefore, a baseline clinical and radiographic assessment is necessary before implant placement to assess pathological or physical alterations in the tissues around implants over time [14].

Clinical diagnosis of peri-implantitis requires peri-implant probing to detect the presence of BoP. When compared to baseline recordings, sites with peri-implantitis exhibit peri-implant mucosal recession, elevated probing depths, and/or clinical indications of inflammation (BoP) [12].

An intra-oral radiograph is necessary to detect increasing marginal bone loss and diagnose peri-implantitis. Marginal bone levels apical to what would be expected after normal remodeling may be utilized to determine the degree of supporting bone loss when prior radiographs are unavailable for comparison. The presence of BoP, probing depths ≥6 mm, and a marginal bone level ≥3 mm apical to the most coronal region of the implant's endosseous component can all be used to diagnose peri-implantitis in the absence of prior examination data [14].

Background on erbium lasers

Er:YAG is a solid active medium made up of yttrium-aluminum crystals that have been purposefully doped with erbium atoms. Er,Cr:YSGG (yttrium scandium gallium garnet) lasers and Er:YAG (yttrium aluminum garnet) lasers are the two different wavelengths in the erbium family of lasers [14].

Dental lasers use an articulated arm, hollow waveguide, or fiber-optic cable to transfer the laser's light to the target tissue. The system is completed with focusing lenses, a cooling system, and additional controls. The composition of an active medium, which can be a gas, a crystal, or a solid-state semiconductor, largely determines the laser's wavelength and other characteristics [6].

Depending on the tissues' water content, absorption of a laser raises the temperature and causes photochemical reactions. When a temperature of 100°C is achieved, evaporation of the water within the tissue occurs, a process termed ablation [8].

Chromophores, which are light absorbers with a particular affinity for particular light wavelengths, are necessary for absorption. Melanin, hemoglobin, and water are the main chromophores in the mouth’s soft tissues, whereas water and hydroxyapatite are the main chromophores in the hard tissues of the teeth. The laser selection process is reliant on the absorption coefficients of various laser wavelengths concerning these key tissue components [15, 16].

## Review

Mechanisms of action of Er:YAG

The Er:YAG laser with a wavelength of 2940 nm and the Er,Cr:YSGG laser with a wavelength of 2780 nm are both utilized in dentistry [17-18]. Since the Er:YAG laser's wavelength of 2,940 nm aligns with the wide absorption band for water, it has the highest absorption of any laser emitting in the near- and mid-infrared spectral region [19]. Consequently, both soft and hard tissues can be treated using erbium lasers as their main chromophore is water, while they also absorb hydroxyapatite to a lesser extent [20]. Soft tissue operations mostly use photothermal interactions, whereas hard tissue procedures primarily use photodisruptive interactions [21-22].

Laser-tissue interaction

Erbium laser wavelengths coincide with water's absorption peak. When water in hard tissue interacts with wavelengths, laser energy is converted to heat and water vapor is created [23-24]. This expands and creates high pressure inside the target tissue, which leads to immediate tissue particle ejection and micro explosions. This is known as thermomechanical ablation and lasers are used in minimally invasive dentistry because of this procedure [25]. Theoretically, the Er:YAG laser's water absorption coefficient is 10,000 times more than that of the CO2 laser and 15,000-20,000 times higher than that of the Nd:YAG (neodymium-doped yttrium aluminum garnet) laser [26].

The terminal portion of the handpiece is held 5-15 mm away from the target tissue when applying erbium lasers to hard tissues in a non-contact mode. In hard tissue, the pulse frequency (Hz), energy (mJ), and power (W) requirements are as follows: Carious tissue: 20-25 Hz, 75-80 mJ, 1.5-2.0 W; Cavity preparation enamel: 25-30 Hz, 150-200 mJ, 3.0-4.5 W; Cavity preparation dentin: 15-20 Hz, 100-120 mJ, 1.8-2 W [27].

Mechanism of action on bacterial biofilm and tissue debridement

The emissions of Er:YAG lasers, which have a wavelength of 2940 nm, are significantly absorbed by water. The brief laser pulses cause fast fluid movement and cavitation events, which can be helpful in breaking up biofilms. The Er:YAG laser exhibits great bactericidal action against periodontal infections because of its strong absorption in water. Bacterial toxins such as lipopolysaccharides are also rendered inactive by their photothermal effects [28].

The current data suggest that: (a) Er:YAG laser seems to be just as effective as hand instrumentation in removing bacterial biofilms from dentin surfaces; and (b) the use of Er:YAG laser produced distinct advantages over the other debridement modalities on titanium surfaces [29].

The Er:YAG laser stimulates/activates the proximal gingival and bone tissues/cells during pocket and bone defect debridement in periodontal surgery. According to in vitro research, low-level Er:YAG laser irradiation causes primary osteoblast-like cells to calcify and gingival fibroblasts periodontal ligament fibroblasts, and osteoblasts to proliferate more [30].

The biostimulation (photobiomodulation) of tissues and cells after irradiation is another intriguing and distinctive feature of lasers; this is an entirely different impact not observed with mechanical treatment [31].

Effects on bone and soft tissue

A comparison of Er and CO2 laser ablation revealed that following Er:YAG laser ablation, the chemical makeup of the bone surface remained almost unaltered. Contrastingly, CO2-laser ablation caused the creation of harmful compounds. An accumulation of red blood cells of different densities was observed dispersing throughout the surface of the treated bone ten minutes following Er:YAG laser ablation. Initial bone healing and general healing events seemed to proceed more quickly in the Er:YAG group than in the CO2 and conventional groups at 6 and 24 hours, as well as 3, 7, and 14 days. Following Er:YAG laser ablation, osteotomy sites showed increased inflammatory cell infiltration, revascularization, and fibroblast and osteoblast proliferation, all of which suggested the creation of active osteoid tissue. The study concluded that the uneven surface structure following Er:YAG laser ablation, which was free of char or smear layers, offered a surface that was conducive to cell attachment, hastening the production and mending of new bone [32].

Er:YAG laser in the treatment of peri-implantitis

The role of Er:YAG lasers in treating peri-implantitis is controversial in the literature. According to studies, Er:YAG lasers performed better than air abrasives, titanium brushes, curettes, and ultrasonic scalers [33]. Nevertheless, several studies have found no benefits to using this type of laser [34, 35].

Er:YAG lasers may improve bacterial decontamination as observed in randomized controlled clinical trials. They observed a statistically significant decrease in pocket depth measurements, but no improvements in clinical attachment level (CAL), gingival index (GI), or radiographic bone level. To validate if Er:YAG laser irradiation offers further therapeutic advantages for peri-implantitis regenerative treatment, the authors concluded that "a larger sample size and longer follow-up are needed" [36].

A high-power diode laser may have some effect on peri-implant pathogens causing peri-implantitis, while Er:YAG laser application shows no significant effect on oral bacteria in the long term, according to the findings of Arisan et al. (2015) [37], Caccianiga et al. (2016) [38], Caccianiga et al. (2021) [39], and Ting et al. (2022) [40].

The effectiveness of oxygen high-level laser therapy (OHLLT) either by itself or in conjunction with an Er:YAG laser was also investigated, mixing the two lasers whenever we needed to enhance bone regeneration. Er:YAG lasers are unable to penetrate deeper than 2 µm into both soft and hard tissues because of the high absorption in water and hydroxyapatite. Instead, because the suggested diode lasers, with a high peak power (2.5 Watt) and low mean power (0.5 Watt), a frequency of 10 KHz, a t-on of 20 microns, and a t-off of 80 microns, can penetrate deeply into oxygenated tissues, OHLLT demonstrated the ability to eliminate periodontal pathogenic bacteria from periodontal and peri-implant pockets and to improve soft and hard tissue healing [41].

There were no statistically significant differences in postoperative bone levels between implants that received only OHLLT and implants with OHLLT + Er:YAG laser, indicating that the Er:YAG laser appears to be ineffective in enhancing bone level gain when compared to OHLLT alone. Our study examined this claim by examining a larger sample of implants with peri-implantitis [42].

Probing pocket defects (PPD) ≥ 6 mm (deep probing depths) prior to surgery were reduced on average to about 3.5 mm at 12 months by employing an Er:YAG laser for implant surface decontamination, removing defect granulomatous tissues, and using grafting therapy for bony defect resolution. Shallow probing depths (less than 6 mm) stayed constant, with a mean PPD of 3.2 mm at 12 months [43].

Safety and challenges

Heat-Related Tissue Damage

The possibility of heat damage to the soft and hard tissues around implants is a major worry with Er:YAG lasers. Long application periods or excessive energy settings might raise local temperatures, harming the soft tissues and surrounding bone. The potential for thermal impacts to impair osseointegration or postpone healing makes them especially troublesome [38].

Undertreatment

Undertreatment can leave behind biofilm, which lowers the efficiency of the intervention, while overtreatment can lead to needless tissue removal. Operator experience is crucial because the delicate balance between adequate disinfection and tissue preservation necessitates accurate control of laser settings [40].

Contraindications

The efficient use of lasers may be restricted by certain clinical situations, such as poor soft tissue health or particular implant materials. Furthermore, high-energy laser irradiation may negatively impact titanium implant surfaces, resulting in microstructural alterations that may compromise their functionality [43].

Technical limitations

The laser's capacity to penetrate and properly disinfect dental implants may be limited by their uneven morphology, especially in areas with elaborate patterns. Furthermore, it is still technically challenging to achieve effective debridement without harming nearby tissues or implants, necessitating exact modifications to energy settings and MDPI delivery methods.

Standardization

The absence of established treatment guidelines is a major obstacle to laser therapy for peri-implantitis. The ideal energy levels, irradiation duration, laser application frequency, and laser types (e.g., diode, Er:YAG, or CO₂) are all up for debate. The development of evidence-based recommendations is made more difficult by this unpredictability, which results in conflicting clinical outcomes across research. For example, some research indicates that laser decontamination is moderately effective, whereas other studies reveal that mechanical debridement alone produces outcomes that are equivalent. This irregularity hinders widespread adoption and poses difficulties for physicians [44].

Differences in Clinical Results

Research on the efficacy of laser treatments in comparison to traditional mechanical therapies has produced conflicting findings. According to one study, lasers may not exhibit higher efficacy but rather produce comparable results, especially in terms of pocket depth reduction and clinical attachment level improvements. This discrepancy prevents broad adoption and emphasizes the necessity of more clinical research to improve laser therapy techniques [43].

Problems With Laser Penetration in Hard-to-Reach Places

Due to its limited penetration in intricate anatomical locations, laser treatment for peri-implantitis frequently encounters difficulties. The efficiency of decontamination may be decreased in some regions, such as undercuts or peri-implant pockets, where direct laser access may not be possible. The size and physical placement of the handpiece further restrict the potential of lasers such as Er:YAG to accomplish adequate energy delivery and tissue contact. According to studies, insufficient access to bacterial biofilms in certain areas may result in lingering illness and poor results [44].

Summary of studies on Er:YAG lasers and other treatments

Table 1 summarizes various studies focused on the effectiveness of Er:YAG lasers and other treatments in managing peri-implantitis and implant decontamination. Hakki et al. (2017) assessed the impact of different cleaning methods on failed titanium implants and found that a combination of air abrasive and citric acid with the Er:YAG laser was the most effective for decontamination [31]. Wang et al. (2021) analyzed the use of lasers in peri-implantitis regenerative surgery, showing that laser treatment helped reduce probing depth (PD) more effectively [34]. Persson et al. (2011) investigated the microbiological and clinical effects of Er:YAG treatment, observing a reduction in Fusobacterium spp. after one month, though bacterial counts rebounded after six months [45]. Caccianiga et al. (2021) compared oxygen high-level laser therapy (OHLLT) alone and in combination with the Er:YAG laser, finding no significant differences in outcomes between the two methods [39]. Lin et al. (2021) highlighted the effectiveness of Er:YAG lasers in removing granulation tissue, calcified deposits, and biofilm from implant surfaces [46]. Clem et al. (2019) found that Er:YAG laser treatment combined with bone grafting resulted in probing pocket depth (PPD) reductions and peri-implant defect fill after one year [44]. Finally, Hattani et al. (2024) reviewed the overall applications of Er lasers in dentistry, emphasizing their efficiency, specificity, and improvement in patient comfort and cost-effectiveness [43].

**Table 1:** Summary of Studies on Er:YAG Laser in Peri-Implantitis Treatment

Study	Objective	Sample	Findings
Hakki et al., 2017 [31]	To assess the impact of different cleaning methods on the surface and temperature of failed titanium implants	27	Air abrasive and citric acid combination and Er:YAG laser groups was the best method for the decontamination of titanium surfaces of failed implant.
Wang et al., 2021 [34]	Analyzing the efficacy of laser for peri-implantitis regenerative surgery	24 peri-implantitis patients	Laser irradiation during peri-implantitis regenerative therapy can help better probing PD reduction
Persson et al., 2011 [45]	Assessment of microbiological and clinical and impacts of Er:YAG treatment of peri-implantitis lesions	Clinical trial of 42 patients	After 1 month *Fusobacterium spp.* were reduced in the laser group. 6 months of data revealed failure of laser in reducing bacterial count.
Caccianiga et al., 2021 [39]	Comparison of OHLLT and OHLLT+ Er laser in peri-implantitis management	210 implants	No statistically significant differences between OHLLT and OHLLT + Er:YAG laser
Lin et al., (2021) [46]	Er:YAG laser-assisted periimplantitis total therapy		Er:YAG laser is effective for removing not only the granulation tissue during surgery, but also the calcified deposit and biofilm on the implant surface
Clem et al., 2019 [44]	Peri-implantitis treatment using Er:YAG laser and bone Grafting	20 patients (23 peri-implants) 1-year evaluation	Radiographs indicated PPD reductions were achieved along with peri-implant defect fill.
Hattani et al., 2024 [43]	Applications of Er lasers in dentistry	Review	Laser proved an efficacious tool to enhance efficiency, specificity, ease, and cost and comfort of the dental treatment.

## Conclusions

The use of Er:YAG lasers to treat peri-implantitis offers both great potential and formidable obstacles with key findings showing that Er:YAG lasers outperform conventional mechanical debridement tools in terms of pocket depth reduction and bacterial decontamination. However, there is still uncertainty regarding their effects on other clinical parameters like clinical attachment level (CAL), gingival index (GI), and radiographic bone levels. Furthermore, the evidence does not always support their efficacy in bone repair and long-term bacterial control.

When operated in ideal circumstances, such as with the right energy settings and water spray, notable increases in tissue healing and bone regeneration were demonstrated. However, this technique is hampered by drawbacks such as heat tissue injury, difficulties reaching complex anatomical areas, and variations in therapeutic procedures.

Furthermore, their regular administration is complicated by technological limitations and the lack of established treatment standards. Future research should focus on creative laser designs, such as more flexible fibers and tips. Additionally, to confirm their function in managing peri-implantitis and their capacity to promote soft and hard tissue regeneration, multicenter randomized controlled trials (RCTs) with bigger sample numbers and longer follow-ups are crucial. 
